# Biventricular strain analysis at 1.5T cardiac MR imaging: preliminary results in volunteers using an iterative SENSE reconstruction with L1 regularization

**DOI:** 10.1186/1532-429X-16-S1-W4

**Published:** 2014-01-16

**Authors:** Peter M Smith, Benjamin H Freed, Bradley D Allen, Bruce S Spottiswoode, Maria Carr, Marie Wasielewski, Karissa Campione, Marius Cordts, Christoph Guetter, Marie-Pierre Jolly, Michaela Schmidt, Mariappan S Nadar, Michael O Zenge, James C Carr, Jeremy D Collins

**Affiliations:** 1 Department of Radiology, Northwestern University, Chicago, Illinois, USA; 2 Healthcare Sector, Siemens AG, Erlangen, Germany; 3 Corporate Technology, Siemens Corporation, Princeton, New Jersey, USA; 4 Department of Cardiology, Northwestern University, Chicago, Illinois, USA; 5 Cardiovascular MR R&D, Siemens Healthcare, Chicago, Illinois, USA

## Background

Changes in myocardial strain have been shown to precede onset of systolic dysfunction in patients with cardiomyopathy. Traditionally performed with echocardiography, acoustic windows can limit strain evaluation. Preliminary work has shown good agreement between myocardial strain derived from deformation field analysis at balanced steady-state free-precession (bSSFP) cine MRI and speckle tracking echocardiography. The application of a novel prototype iterative SENSE reconstruction with L1 regularization (IR-SENSE) to highly accelerated segmented bSSFP cine acquisitions can maintain or improve the effective temporal resolution with a shorter breath-hold. The purpose of this study is to evaluate left- and right-ventricular stain analysis from bSSFP cine acquisitions using conventional GRAPPA and highly accelerated T-PAT with IR-SENSE. We hypothesize that myocardial strain parameters derived from accelerated T-PAT cine acquisitions with IR-SENSE are similar to those obtained using conventional segmented bSSFP with comparable effective temporal resolutions.

## Methods

9 healthy volunteers (6 males, 44.3 ± 13.5 yrs) underwent imaging at 1.5 T (MAGNETOM Aera, Siemens AG, Healthcare Sector, Erlangen, Germany). The following 5 pulse sequences were acquired in the short axis and 4-chamber orientations: GRAPPA factor 2 standard bSSFP cine with 5 and 14 views per segment (Seg NIR), and T-PAT factor 4 bSSFP cine with 2, 8, and 14 views per segment reconstructed using a prototype IR-SENSE algorithm (Seg IR) (Table 1). Strain values were derived from deformation field analysis on prototype software (Siemens Corporate Technology, Princeton, NJ). LV global and RV lateral wall longitudinal strains were obtained from 4-chamber cine acquisitions. LV circumferential and radial strains were obtained from short-axis acquisitions for the base, mid-chamber, and apex. Average and peak strains were compared using a two-sided student's t-test.

**Table 1:** Cardiac MRI sequence parameters.

Sequence abbreviation	In-Plane resolution (mm × mm)	Slice thickness (mm)	Views per segment	Acceleration technique and factor	Effective temporal resolution (msec)	Acquisition time (sec)
2-Seg IR	1.9 × 1.8	6	2	T-PAT SENSE factor 4	5.5	19

8-Seg IR	1.9 × 1.8	6	8	T-PAT SENSE factor 4	22	6

14-Seg IR	1.9 × 1.8	6	14	T-PAT SENSE factor 4	38.6	4

5-Seg NIR	1.5 × 2.1	6	5	iPAT GRAPPA factor 2	12.5	17

14-Seg NIR	1.5 × 1.5	6	14	iPAT GRAPPA factor 2	39.2	8.4

IR: iterative reconstruction using k-t SENSE

## Results

All T-PAT 4 cine bSSFP acquisitions were successfully reconstructed using the IR-SENSE algorithm. Average and peak systolic strains by region and sequence are shown in Table 2. Longitudinal strains were similar across all acquisitions (p > 0.05), with a trend towards greater strain values for 5-Seg NIR and 2-Seg IR acquisitions. LV radial and circumferential strains were regionally similar except for differences between 5-Seg NIR and 8-Seg IR LV radial strain in the mid-chamber and 5-Seg NIR and 14-Seg NIR LV circumferential strain in the apical chamber (p < 0.05).

**Table 2:** Segmental end systolic strain results (average, peak; expressed as a percentage) by imaging sequence and cardiac region.

Sequence Abbreviation	LV Longitudinal	RV Longitudinal Free Wall	LV Radial Basal Chamber	LV Radial Mid Chamber	LV Radial Apical Chamber	LV Circum Basal Chamber	LV Circum Mid Chamber	LV Circum Apical Chamber
2-Seg IR	-17.1, -26.1	-17.2, -24.9	37.7, 56.9	41.2, 62.7	32.6, 47.1	-20.3, -24.7	-22.3, -27.4	-25.2, -28.2

8-Seg IR	-16.0, -23.3	-17.1, -23.3	29.5, 42.8	36.1, 48.1	34.5, 50.4	-19.4, -24.2	-20.4, -24.1	-22.5, -25.8

14-Seg IR	-15.0, -22.7	-16.4, -23.0	36.4, 61.1	41.5, 59.9	38.3, 54.1	-18.9, -24.0	-20.8, -24.3	-23.7, -26.4

5-Seg NIR	-16.6, -24.8	-19.5, -26.4	40.8, 62.0	50.2, 74.7	29.7, 41.9	-19.8, -25.5	-21.9, -26.1	-20.7, -23.4

14-Seg NIR	-16.8, -24.0	-19.6, -26.0	41.0, 64.5	47.7, 69.1	41.7, 63.3	-19.7, -24.6	-21.9, -26.0	-26.6, -29.6

## Conclusions

This preliminary study demonstrates that myocardial strains derived from deformation field analysis on highly accelerated bSSFP cine acquisitions with IR-SENSE are similar to strains derived from conventionally reconstructed segmented bSSFP cine sequences with similar effective temporal resolutions.

## Funding

None.

